# Clinical characteristics and risk factors for *Mycoplasma pneumoniae* pneumonia in children

**DOI:** 10.3389/fped.2024.1438631

**Published:** 2024-12-23

**Authors:** Xueqi Zhao, Jiajia Lv, Min Wu, Qun Wu

**Affiliations:** ^1^ Department of Pediatrics, Ruijin Hospital Affiliated to Shanghai Jiaotong University School of Medicine, Shanghai, China; ^2^ Wenzhou Institute, University of Chinese Academy of Sciences, Wenzhou, China

**Keywords:** *Mycoplasma pneumoniae* pneumonia, non-*Mycoplasma* pneumonia, inflammatory cytokines, lactic dehydrogenase, immune response, long COVID-19

## Abstract

**Background:**

*Mycoplasma pneumoniae* (*M. pneumoniae*) is one of the most common pathogens of community-acquired pneumonia (CAP) in children. Although *Mycoplasma pneumoniae* pneumonia (MPP) is considered a self-limiting disease, severe MPP (SMPP) occurs in some cases. This study aims to analyze clinical features of MPP and to explore predictive indicators in the early stage of *M. pneumoniae* infection.

**Methods:**

We retrospectively enrolled patients with MPP and non-MPP (NMPP) hospitalized to the Department of Pediatrics, Ruijin Hospital, Shanghai Jiao Tong University School of Medicine from 2023 to 2024. A total of 757 children with CAP were divided into MPP group and NMPP group. Patients with MPP included SMPP group and mild MPP (MMPP) group. Demographic and clinical characteristics as well as laboratory and imaging tests were deemed to be baseline data within 24 h after admission. We compared differences between MPP group and NMPP group as well as SMPP group and MMPP group. To exclude the impacts of age and gender, analysis of covariance and Logical regression was used to account for the baseline differences in the probability between MPP group and NMPP group, SMPP group and MMPP group. Logistic regression analysis was used to screen markers as potential early clinical predictors. ROC curves were applied to estimate the diagnostic and predictive value of different indicators for SMPP group.

**Results:**

Among the 757 cases of CAP, 464 cases were MPP group and 226 cases were SMPP group. There were significant differences in hospital stay and fever duration between the MPP and NMPP groups. Compared to NMPP group, MPP group exhibited higher levels of platelet count (PLT), heparin-binding protein (HBP), erythrocyte sedimentation rate (ESR), immunoglobulin G (IgG) (*P* < 0.05). The levels of C-reactive protein (CRP), erythrocyte sedimentation rate (ESR), serum ferritin (SF), prothrombin time (PT), fibrinogen (Fg), interleukin-5 (IL-5) and Gamma interferon (IFN-γ) were significantly increased in SMPP group compared to MMPP group. PT, Fg, SF, IL-5 and IFN-γ were independent risk factors for SMPP group. Significantly, IL-5 and IFN-γ served as reliable predictive indices of SMPP.

**Conclusions:**

Notable differences were observed in both clinical characteristics and serum inflammatory markers between the MPP group and the NMPP group, as well as between the SMPP group and the MMPP group. Consequently, PT, Fg, SF, IL-5 and IFN-γ hold the potential to be employed as efficacious predictors for SMPP.

## 1 Introduction

*Mycoplasma pneumoniae* (*M. pneumoniae*) is the most common seen pathogen of community-acquired pneumonia (CAP) in children. *M. pneumoniae* pneumonia (MPP) is characterized by pulmonary interstitial disease that can injure other organs through local respiratory infection (1), which can be seen in any season throughout the year. The infection rate of MPP in children >5 years old can reach as high as 50% (2), constituting 30% of pediatric CAP (3, 4). Consequently, MPP was very widespread in pediatrics. In recent years, non-pharmaceutical interventions against COVID-19 and its complications (long COVID-19) drastically curbed the transmission of *M. pneumoniae* (5). However, since the latter half of 2023, a substantial increase in the number of children with MPP has been witnessed globally, as has been the case in Shanghai, China, which might be associated with long COVID-19 (6, 7). Pediatric patients with *M. pneumoniae* infection presented with fever, cough, wheezing, dyspnea and other symptoms and some patients, especially older children, may progress to severe MPP (SMPP), which may be caused by direct pathogen invasion, *M. pneumoniae* resistance, abnormal immune inflammatory responses and mixed infections (8). Additionally, SMPP often induces damage to extrapulmonary tissues and organs, giving rise to conditions like encephalitis, nephritis, hepatitis or even multiple organ failure. This not only impairs children's health, but also imposes an augmented economic burden on families and society. Currently, MPP lacks specific clinical manifestations in the early stage and it is challenging to distinguish lung damage caused by other pathogens. Therefore, accurate identification of MPP and correct assessment of its severity at an early stage will provide strong evidence for clinical diagnosis and treatment.

*M. pneumoniae* proliferates in respiratory epithelial cells by binding P1 protein to cilia, stimulates the production of proinflammatory cytokines in airway mucosa, induces cellular inflammatory responses and tissue damage and ultimately leads to changes in host immune function (9, 10). The imbalance of Th1/Th2 function following *M. pneumoniae* infection has been considered as an crucial immunological mechanism of MPP. Therefore, exploration of the blood biochemical and inflammatory predictors in children with MPP holds significant value in guiding clinical treatment. The objective of this study was to examine the variations in clinical manifestations and laboratory tests results among the MPP group, non-MPP (NMPP) group, SMPP group and mild MPP (MMPP) group. Additonally, we aimed to identify the early predictive markers of SMPP group for the purpose of early and precise identification in clinical practice and to establish a foudation for the early treatment of the MPP group.

## 2 Methods

### 2.1 Study subjects

**Figure 1:**
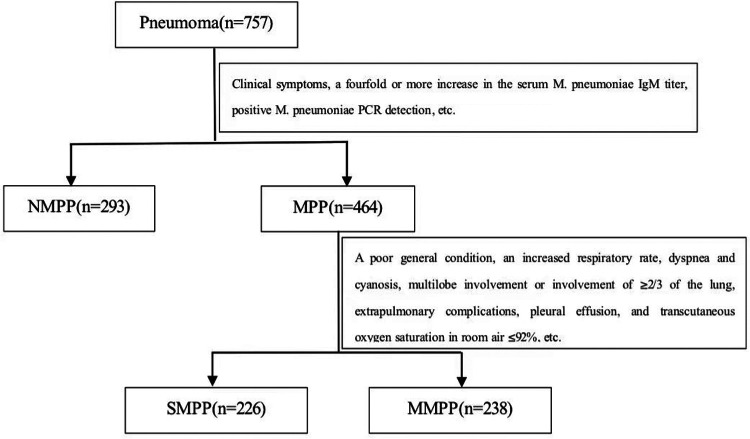
Study flow chart. 757 children with pneumonia hospitalized were divided to NMPP group and MPP group by Clinical symptoms, a fourfold or more increase in the serum *M. pneumoniae* IgM titer, positive *M. pneumoniae* PCR detection, etc. MPP group were divided into SMPP group and MMPP group according to the standard which were a poor general condition, an increased respiratory rate, dyspnea and cyanosis, multilobe involvement or involvement of ≥2/3 of the lung, extrapulmonary complications, pleural effusion, and transcutaneous oxygen saturation in room air ≤92%, etc.

We retrospectively collected clinical data of 757 children with pneumonia hospitalized in the Department of Pediatrics, Ruijin Hospital, Shanghai Jiao Tong University School of Medicine from January 2023 to February 2024 were selected, including 464 children with MPP (including 226 cases of SMPP and 238 cases of MMPP) and 293 cases of NMPP. Severe pneumonia was defined as pneumonia plus one of the following characteristics: (1) a poor general condition, (2) an increased respiratory rate (infants >70 breaths/min and older children >50 breaths/min), (3) dyspnea and cyanosis, (4) multilobe involvement or involvement of ≥2/3 of the lung, (5) extrapulmonary complications, (6) pleural effusion, and (7) transcutaneous oxygen saturation in room air ≤92% (11). The inclusion criteria of MPP group are as follows: (1) age 1–15 years old; (2) meet the diagnostic criteria of CAP, have acute respiratory tract infection symptoms, chest imaging findings showed new infiltrate or consolidation with or without pleural effusion; (3) a fourfold or more increase in the serum *M. pneumoniae* IgM titer during the recovery period (but not the acute period), or a positive *M. pneumoniae* PCR detection in nasopharyngeal swab; (4) patients with the concomitant viral infection or specific infection of other pathogens were excluded (12). The inclusion criteria of NMPP group are as follows: (1) age 1–15 years old; (2) meet the diagnostic criteria of CAP; (3) children with confirmed infections of influenza A, influenza B, human metapneumovirus, adenovirus, respiratory syncytial virus, parainfluenza and other viruses were included; (4) children with definite or suspected *M. pneumoniae* or chlamydia infection were excluded (13). The detailed study design was demonstrated in Figure 1*.* Demographic and clinical features, laboratory and imaging findings of all children included in the study were collected within 24 h after admission, including chief complaint, length of hospital stays, fever, fever duration, cough, wheezing, chest radiography, lung computed tomography (CT), white blood cell (WBC), neutrophil count (NEU), C-reactive protein (CRP), procalcitonin (PCT), erythrocyte sedimentation rate (ESR), serum ferritin (SF), heparin-binding protein (HBP), Tumor necrosis factor (TNF), alanine aminotransferase (ALT), aspartate aminotransferase (AST), lactic dehydrogenase (LDH), blood platelet count (PLT) and coagulation indexes. Fever duration refers to the number of days when the body temperature exceeds 37.8 degrees Celsius at least once. The study was approved by the Ethics Committee of The Affiliated Ruijin Hospital, Shanghai Jiao Tong University School of Medicine [(2024) LRC No. (122)] with a waiver of informed consent, and this study was conducted in accordance with relevant guidelines and regulations.

### 2.2 Inflammatory factors in blood

The fasting venous whole blood (10 ml) was collected from all the children in the day after admission, and the serum was separated after centrifugation at 3,000 r/min and stored in a −80℃ refrigerator. SF were detected by automatic chemiluminescence immunoanalyser (Beckman DXI800). The concentrations of the cytokines interleukin-4 (IL-4), interleukin-5 (IL-5), Gamma interferon (IFN-γ), tumor necrosis factor (TNF) were determined using double-antibody sandwich enzyme-linked immunosorbent assay (ELISA, Human Cytokine Panel, Qingdao Riskell Biotechnology Co., LTD, BD FACS Canto II). The percentage of CD3^+^, CD4^+^, CD8^+^, NK and CD19^+^ cells were detected by flow cytometry (Gold standard method, BD FACS Canto II). ALT and AST were detected by velocity method (Beckman AU5821). The morning after hospitalized, 2 ml of venous whole blood was taken on an empty stomach, and the blood was centrifuged at 2,000 r/min for 10 min, followed by a colloidal gold method (Bioneovan Co., Ltd.) for the detection of mycoplasma pneumoniae IgM antibodies in the children. All experimental procedures were carried out strictly accordance with the procedure's instructions.

### 2.3 Statistical analysis

SPSS software (19th Edition) was used for statistical analysis. Data distribution in all groups was determined by the Kolmogorov–Smirnov test. The normal distribution data was expressed as mean ± standard deviation (SD). Oneway ANOVA or independent-samples *T*-test was used to process these data. Mann–Whitney *U*-test was used for comparison between these data. Categorical data were shown as frequencies or percentages. We used Pearson chi-square test or Fisher's exact test to analyze differences between categorical variables. To exclude the impacts of age and gender, analysis of covariance and Logical regression was used to account for the baseline differences in the probability between MPP and NMPP groups, SMPP and MMPP groups. Meanwhile, the receiver operating characteristic (ROC) curve estimate the worth of indicators in predicting and diagnosing SMPP group. *P* < 0.05 is defined as a statistically significant difference.

## 3 Results

### 3.1 Demographic and clinical characteristics

A total of 757 children who met the diagnostic criteria for bronchopneumonia were collected, and there were 464 with MPP and 293 with NMPP. The average age of the MPP group is 1.14 years older than that of the NMPP group (6.97 ± 2.81 years vs. 5.83 ± 3.43 years, *P* < 0.001). In contrast to NMPP group the days of fever duration (6.1 ± 3.37 days vs. 4.93 ± 3.34 days, *P* < 0.001) were longer and the percentage of fever increases (91.59% vs. 84.23%, *P* = 0.006) in MPP group. There were also more cases of extrapulmonary symptom (6.03% vs. 2.35%, *P* = 0.018) and pulmonary consolidation (12.30% vs. 2.35%, *P* = 0.004) than in the NMPP group. The extrapulmonary manifestations of MPP group were mainly rash, liver function damage, myocardial damage and abnormal coagulation index. There were no obvious differences in hospital stays, cough and wheezing between the two groups (Table 1).

**Table 1:** Comparison of demographic and clinical characteristics between MPP and NMPP groups after analysis of covariance and logical regression.

General information	MPP (464)	NMPP (293)	*P*
Sex (male/female)	215/249	160/133	0.027
Age (years)	6.97 ± 2.81	5.83 ± 3.43	0.000**
Fever duration (days)	6.1 ± 3.37	4.93 ± 3.34	0.000**
Hospital stays (days)	6.26 ± 2.17	5.94 ± 2.1	0.195
Fever, *n* (%)	425 (91.59)	251 (84.23)	0.006**
Cough, *n* (%)	444 (95.69)	250 (83.89)	0.992
Wheezing, *n* (%)	13 (2.80)	10 (3.36)	0.663
Extrapulmonary symptom, *n* (%)	28 (6.03)	7 (2.35)	0.018*
Pulmonary consolidation, *n* (%)	57 (12.30)	14 (4.80)	0.004**

The data were the baseline measurement results made within 24 h after admission. *P* < 0.05 indicates statistical significance.

MPP, *Mycoplasma pneumoniae* pneumonia; NMPP, non-*Mycoplasma pneumoniae* pneumonia.

*P < 0.05.

**P < 0.01.

### 3.2 Results of blood parameters between the MPP and NMPP groups

The difference of inflammation index between MPP group and NMPP group was listed in Table 2. The levels of PLT, HBP and ESR in MPP group were apparently higher than those in NMPP group; conversely, the level of PCT was lower. No differences were found in white blood cell (WBC), NEU, CRP, LDH, SF and TNF between MPP and NMPP groups (Table 2).

**Table 2:** Comparison of blood parameters between MPP and NMPP groups after analysis of covariance.

Clinical index	MPP		NMPP		*P*
	*n*	$\bar{x}\pm s$	*n*	$\bar{x}\pm s$	
WBC (×10^9^/L)	463	8.42 ± 26.77	293	7.05 ± 4.19	0.253
NEU (×10^9^/L)	450	4.37 ± 3.7	289	3.76 ± 4.26	0.159
PLT (×10^9^/L)	463	310.46 ± 105.09	293	272.72 ± 114.71	0.000**
CRP (mg/L)	463	16.07 ± 18.57	291	13.85 ± 16.36	0.264
HBP (ng/ml)	453	83.03 ± 71.87	272	59.2 ± 62.31	0.000**
ESR (mm/h)	453	21.9 ± 15.52	272	15.76 ± 14.63	0.000**
PCT (ng/ml)	461	0.14 ± 0.3	285	0.21 ± 0.44	0.046
LDH (IU/L)	193	280.11 ± 84.72	197	308.57 ± 130.3	0.102
SF (ng/ml)	339	92.93 ± 52.1	117	98.32 ± 88.31	0.161
TNF (pg/ml)	330	7.21 ± 9.88	92	6.95 ± 7.23	0.873

WBC, white blood cell, NEU, neutrophil count; PLT, blood platelet count, CRP, C-reactive protein; HBP, heparin-binding protein, ESR, erythrocyte sedimentation rate; PCT, procalcitonin; LDH, lactic dehydrogenase; SF, serum ferritin; TNF, tumor necrosis factor.

**P < 0.01.

The levels of cellular and humoral immune reactions between MPP group and NMPP group were detected (Table 3). The result showed that the level of IgG (9.82 ± 2.4 g/L vs. 8.71 ± 2.47 g/L, *P* = 0.001) in the MPP group was apparently higher than those in the NMPP children, on the contrary, the level of CD3^+^ (68.03 ± 9.98 vs. 69.23 ± 10.02, *P* = 0.008) was lower, meanwhile, there were no difference in the percentages of CD3^+^CD4^+^, CD3^+^CD8^+^, CD4^+^ CD8^+^, NK cell and CD19^+^ and IgA, IgM between the two groups (Table 3).

**Table 3:** Comparison of cellular immunity and humoral immunity between MPP and NMPP groups after analysis of covariance.

Clinical index	MPP		NMPP		*P*
	*n*	$\bar{x}\pm s$	*n*	$\bar{x}\pm s$	
IgG	423	9.82 ± 2.4	268	8.71 ± 2.47	0.001**
IgA	423	1.47 ± 0.68	268	1.53 ± 6.14	0.380
IgM	424	2.01 ± 10.38	268	1.26 ± 2.31	0.194
CD3^+^	402	68.03 ± 9.98	253	69.23 ± 10.02	0.008
CD3^+^CD4^+^	402	36.13 ± 9.31	253	36.36 ± 8.92	0.500
CD3^+^CD8^+^	402	27.21 ± 8.46	253	28.22 ± 13.09	0.119
CD4^+^/CD8^+^	401	1.46 ± 0.61	253	1.51 ± 0.75	0.718
NK	110	11.45 ± 18.32	143	9.71 ± 6.24	0.151
CD19^+^	109	19.64 ± 8.14	143	21.44 ± 21.54	0.946

The data were the baseline measurement data made within 24 h after admission. *P* < 0.05 indicates statistical significance.

IgG, immunoglobulin G; IgA, immunoglobulin A; IgM, immunoglobulin M.

**P < 0.01.

Multiple organ damage includes coagulation dysfunction, liver damage and myocardial damage (Table 4). Among them, the levels of TT, CTnI and AST in the MPP group were apparently lower than those in the NMPP group. No significant differences were found in APTT, PT, INR, Fg, D-D, FDP, CK, CK-MB and ALT between MPP and NMPP groups (Table 4).

**Table 4:** Comparison of biochemical indicators between MPP and NMPP groups after analysis of covariance.

Clinical index	MPP		NMPP		*P*
	*n*	$\bar{x}\pm s$	*n*	$\bar{x}\pm s$	
APTT	448	31.77 ± 3.53	280	31.82 ± 4.51	0.682
PT	448	11.94 ± 1.06	280	12.18 ± 6.61	0.279
INR	448	1.24 ± 4.35	280	1.01 ± 0.09	0.299
TT	447	16.13 ± 1.37	280	17.16 ± 6.29	0.007**
Fg	448	3.61 ± 0.83	279	3.47 ± 1.05	0.460
D-D	453	0.44 ± 1.00	283	0.57 ± 1.52	0.117
FDP	454	2.67 ± 10.33	283	2.56 ± 3.99	0.822
CK	117	172.65 ± 442.56	174	152.02 ± 231.64	0.997
CK-MB	462	1.62 ± 2.50	291	1.86 ± 1.83	0.393
CTnI	462	2.74 ± 3.95	291	4.95 ± 12.83	0.003**
ALT	464	19.99 ± 44.99	290	17.4 ± 13.79	0.287
AST	464	31.85 ± 23.52	290	37 ± 18.10	0.032*

The data were the baseline measurement results made within 24 h after hospital admission. *P* < 0.05 indicates statistical significance.

APTT, activated prothrombin time; PT, prothrombin time; INR, international normalized ratio; TT, thrombin time; Fg, fibrinogen; D-D, d-dimer; FDP, fibrin degradation products; CK, creatine kinase; CK-MB, creatine kinase isoenzyme; CTnI, cardiac troponin I; ALT, alanine aminotransferase; AST, aspartate aminotransferase.

*P < 0.05.

**P < 0.01.

We performed comparison of Th1/Th2 function between MPP and NMPP groups (Table 5), the levels of EOS, IgE, IL-4, IL5 and IFN-γ were no significant differences between MPP and NMPP groups (Table 5).

**Table 5:** Comparison of Th1/Th2 function between MPP and NMPP groups after analysis of covariance.

Clinical index	MPP		NMPP		*P*
	*n*	$\bar{x}\pm s$	*n*	$\bar{x}\pm s$	
EOS	454	0.12 ± 0.17	292	0.11 ± 0.18	0.263
IgE	423	298.15 ± 496.81	268	255.53 ± 412.23	0.520
IL4	381	3.12 ± 2.7	123	2.87 ± 1.45	0.337
IL5	381	7.99 ± 47.49	122	4.3 ± 4.66	0.424
IFN-γ	379	73.38 ± 284.76	118	69.39 ± 326.96	0.854

The data were the baseline measurement results made within 24 h after hospital admission. *P* < 0.05 indicates statistical significance.

EOS, eosinophils; IgE, immunoglobulin E; IL-4, interleukin-4; IL-5, interleukin-5; IFN-γ, Gamma interferon.

### 3.3 Demographic and clinical characteristics between the SMPP and MMPP groups

The SMPP group was on average 1.43 years older than the MMPP group (7.7 ± 2.59 years vs. 6.27 ± 2.85 years, *P* < 0.001). The days of hospital stays (7.4 ± 2.11 days vs. 5.19 ± 1.6 days, *P* < 0.001) in SMPP group were longer than MMPP group. In addition, we found that the extrapulmonary symptom (9.73% vs. 2.55%, *P* = 0.003) and pulmonary consolidation (24.34% vs. 0.84%, *P* < 0.001) of SMPP group were significantly increased than those in MMPP group. There were no obvious differences in sex, fever duration, fever, cough, wheezing, oxygen therapy, mixed infection and gamma globulin between the two groups (Table 6).

**Table 6:** Comparison of demographic and clinical characteristics between the SMPP and MMPP groups after analysis of covariance and logical regression.

General information	SMPP (226)	MMPP(238)	*P*
Sex (male/female)	103/123	112/126	0.791
Age (years)	7.7 ± 2.59	6.27 ± 2.85	0.000**
Fever duration (days)	6.51 ± 2.87	5.89 ± 3.64	0.061
Hospital stays (days)	7.4 ± 2.11	5.19 ± 1.6	0.000**
Fever, *n* (%)	213 (94.25)	212 (89.08)	0.054
Cough, *n* (%)	221 (97.79)	223 (94.89)	0.445
Wheezing, *n* (%)	5 (2.21)	8 (3.40)	0.981
Extrapulmonary symptom, *n* (%)	22 (9.73)	6 (2.55)	0.003**
Pulmonary consolidation, *n* (%)	55 (24.34)	2 (0.84)	0.000**
Oxygen therapy, *n* (%)	20 (8.85)	0 (0.00)	0.994
Mixed infection, *n* (%)	44 (19.50)	55 (23.11)	0.562
Gamma globulin, *n* (%)	25 (11.10)	0 (0.00)	0.994

The data were the baseline measurement results made within 24 h after admission. *P* < 0.05 indicates statistical significance.

MMPP, mild *Mycoplasma pneumoniae* pneumonia; SMPP, severe *Mycoplasma pneumoniae* pneumonia.

**P < 0.01.

The differences of infection index between the SMPP and MMPP groups were compared (Table 7). The level of PLT in SMPP group was apparently lower than those in MMPP group, conversely, CRP, ESR and SF were higher. No differences were found in WBC, NEU, HBP, PCT, LDH and TNF between SMPP and MMPP groups (Table 7).

**Table 7:** Comparison of blood parameters between the SMPP and MMPP groups after analysis of covariance.

Clinical index	SMPP		MMPP		*P*
	*n*	$\bar{x}\pm s$	*n*	$\bar{x}\pm s$	
WBC (×10^9^/L)	226	6.53 ± 2.64	238	10.2 ± 37.2	0.280
NEU (×10^9^/L)	223	4.46 ± 4.49	234	4.23 ± 2.72	0.837
PLT (×10^9^/L)	226	293.22 ± 94.47	238	327.15 ± 111.88	0.006**
CRP (mg/L)	226	18.66 ± 18.41	238	13.58 ± 18.39	0.016*
HBP (ng/ml)	220	80.94 ± 69.33	233	85 ± 74.28	0.688
ESR (mm/h)	222	24.64 ± 16.31	231	19.27 ± 14.27	0.000**
PCT (ng/ml)	223	0.15 ± 0.36	238	0.13 ± 0.23	0.091
LDH (IU/L)	105	279.14 ± 85.48	88	281.26 ± 84.27	0.398
SF (ng/ml)	161	101.15 ± 58.74	178	85.5 ± 44.13	0.040*
TNF (pg/ml)	169	8.02 ± 12.21	161	6.36 ± 6.53	0.143

The data were the baseline measurement results made within 24 h after admission. *P* < 0.05 indicates statistical significance.

WBC, white blood cell, NEU, neutrophil count; PLT, blood platelet count, CRP, C-reactive protein; HBP, heparin-binding protein, ESR, erythrocyte sedimentation rate; PCT, procalcitonin; LDH, lactic dehydrogenase; SF, serum ferritin; TNF, tumor necrosis factor.

*P < 0.05.

**P < 0.01.

The levels of cellular and humoral immune response between SMPP and MMPP groups were determined (Table 8). The result showed that the levels of the percentages of CD3^+^ and CD3^+^CD8^+^ in SMPP group were apparently lower than those in MMPP group, however, there were no differences in IgG, IgA, IgM, the percentages of CD3^+^CD4^+^, CD4^+^/CD8^+^, NK cell and CD19^+^ between the two groups (Table 8).

**Table 8:** Comparison of cellular immunity and humoral immunity between SMPP and MMPP groups after analysis of covariance.

Clinical index	SMPP		MMPP		*P*
	*n*	$\bar{x}\pm s$	*n*	$\bar{x}\pm s$	
IgG	200	10.03 ± 2.29	223	9.63 ± 2.49	0.598
IgA	199	1.54 ± 0.67	224	1.41 ± 0.68	0.658
IgM	200	1.39 ± 0.52	224	2.57 ± 14.26	0.410
CD3^+^	192	67.38 ± 10.38	210	68.63 ± 9.59	0.008**
CD3^+^CD4^+^	192	35.92 ± 9.19	210	36.32 ± 9.44	0.477
CD3^+^CD8^+^	192	26.43 ± 6.24	210	27.92 ± 10.03	0.005**
CD4^+^/CD8^+^	192	1.43 ± 0.5	209	1.49 ± 0.69	0.967
NK	61	10.74 ± 8.78	49	12.34 ± 25.78	0.686
CD19^+^	61	19.57 ± 7.92	48	19.74 ± 8.49	0.640

The data were the baseline measurements made within 24 h after admission. *P* < 0.05 indicates statistical significance.

IgG, immunoglobulin G; IgA, immunoglobulin A; IgM, immunoglobulin G.

**P < 0.01.

Multiple organ damage includes coagulation dysfunction, liver damage and myocardial damage (Table 9). Among them, the levels of PT and Fg in SMPP group were significantly higher than those of MMPP group, while the levels TT, CK-MB were lower. No significant differences were found in APTT, INR, D-D, FDP, CK, CTnI, ALT and AST between SMPP and MMPP groups (Table 9).

**Table 9:** Comparison of biochemical indicators between SMPP and MMPP groups after analysis of covariance.

Clinical index	SMPP		MMPP		*P*
	*n*	$\bar{x}\pm s$	*n*	$\bar{x}\pm s$	
APTT	225	32.09 ± 3.31	229	31.5 ± 3.7	0.211
PT	225	12.2 ± 1.01	229	11.7 ± 1.06	0.001**
INR	225	1.06 ± 0.08	229	1.42 ± 6.08	0.494
TT	225	15.82 ± 1.2	228	16.46 ± 1.44	0.000**
Fg	225	3.87 ± 0.75	229	3.36 ± 0.83	0.000**
D-D	224	0.44 ± 0.73	229	0.43 ± 1.21	0.605
FDP	225	3.09 ± 12.9	229	2.26 ± 6.94	0.217
CK	64	129.48 ± 124.66	53	224.77 ± 642.68	0.084
CK-MB	224	1.35 ± 0.74	238	1.87 ± 3.39	0.026*
CTnI	224	2.29 ± 4.04	238	3.17 ± 3.83	0.065
ALT	226	21.92 ± 63.26	238	18.16 ± 12.2	0.392
AST	226	31.06 ± 27.67	238	32.59 ± 18.78	0.972

The data were the baseline measurements made within 24 h after hospital admission. *P* < 0.05 indicates statistical significance.

APTT, activated prothrombin time; PT, prothrombin time; INR, international normalized ratio; TT, thrombin time; Fg, fibrinogen; D-D, d-dimer; FDP, fibrin degradation products; CK, creatine kinase; CK-MB, creatine kinase isoenzyme; CTnI, cardiac troponin I; ALT, alanine aminotransferase; AST, aspartate aminotransferase.

*P < 0.05.

**P < 0.01.

We performed comparison of Th1/Th2 function between the SMPP group and MMPP group (Table 10), which showed that the levels of IL-5 (13.62 ± 61.82 pg/ml vs. 3.89 ± 2.83 pg/ml, *P* = 0.024) and IFN-γ (129.39 ± 369.79 pg/ml vs. 23.77 ± 69.7 pg/ml, *P* < 0.001) in the SMPP group were significantly higher than those in the MMPP group, but no difference in EOS, IgE and IL-4 levels between these two groups (Table 10).

**Table 10:** Comparison of Th1/Th2 function between the SMPP and MMPP groups after analysis of covariance.

Clinical index	SMPP		MMPP		*P*
	*n*	$\bar{x}\pm s$ (M ± QR)	*n*	$\bar{x}\pm s$ (M ± QR)	
EOS	225	0.11 ± 0.17	232	0.13 ± 0.18	0.444
IgE	200	329.73 ± 547.18	223	269.83 ± 446.2	0.356
IL-4	195	3.19 ± 2.89	186	3.04 ± 2.49	0.655
IL-5	195	13.62 ± 61.82	186	3.89 ± 2.83	0.024*
IFN-γ	194	129.39 ± 369.79	185	23.77 ± 69.7	0.000**

The data were the baseline measurements made within 24 h after hospital admission. *P* < 0.05 indicates statistical significance.

EOS, eosinophils; IgE, immunoglobulin E; IL-4, interleukin-4; IL-5, interleukin-5; IFN-γ, Gamma interferon.

*P < 0.05.

**P < 0.01.

### 3.4 Predictive value of independent correlation factors for SMPP group

**Figure 2:**
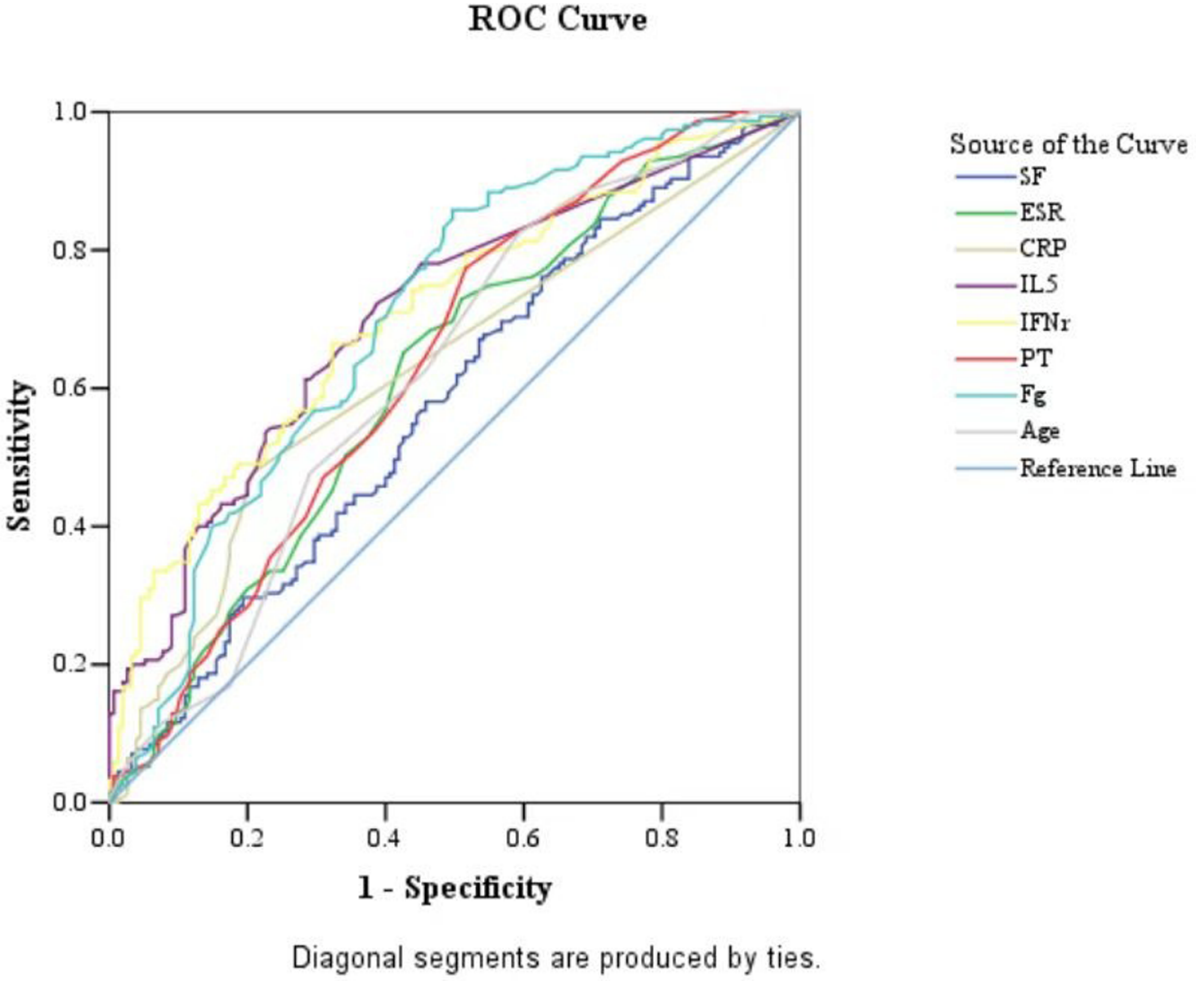
ROC curves of SF, ESR, CRP, IL-5, IFN-γ, PT, Fg and Age values in the SMPP group. The MMPP group as a reference.

We examined the predictive values for SMPP group ROC curves showed that the Fg (AUC = 0.704, sensitivity = 85.8%, specificity = 50.3%) above 3.155 g/L was good predictor for SMPP group (Table 11 and Figure 2). IL-5 (AUC = 0.706, sensitivity = 72.3%, specificity = 61.3%) above 2.85 pg/ml and IFN-γ (AUC = 0.710, sensitivity = 66.5%, specificity = 67.7%) above 10.05 mg/ml were the second useful biomarker. PT had the less predictive value for SMPP group (Table 11 and Figure 2). SF (AUC = 0.577, sensitivity = 67.1%, specificity =46.5%) above 76.75 ng/ml, ESR (AUC = 0.616, sensitivity = 65.2%, specificity = 57.4%) above 20.5 mm/h and CRP (AUC = 0.629, sensitivity = 47.7%, specificity = 79.4%) above 10.5 mg/L were the useful biomarker too. Age above 5.5 years was the least predictive value because they have high sensitivity, but low specificity (Table 11 and Figure 2).

**Table 11:** Predictive value of independent correlation factors for SMPP group.

Independent factors	Cut-off value	AUC	SE	95% CI		Sensitivity	Specificity	*P*
SF (ng/ml)	76.75	0.577	0.032	0.514	0.641	0.671	0.465	0.019*
ESR (mm/h)	20.50	0.616	0.032	0.554	0.679	0.652	0.574	0.000**
CRP (mg/L)	10.5	0.629	0.032	0.566	0.691	0.477	0.794	0.000**
IL5 (pg/ml)	2.85	0.706	0.029	0.648	0.764	0.723	0.613	0.000**
IFN-r (mg/ml)	10.05	0.710	0.029	0.653	0.767	0.665	0.677	0.000**
PT (s)	11.35	0.636	0.031	0.575	0.698	0.774	0.484	0.000**
Fg (g/L)	3.155	0.704	0.030	0.646	0.762	0.858	0.503	0.000**
Age (years)	5.500	0.623	0.032	0.561	0.686	0.826	0.406	0.000**
Combined ROC curve	0.524	0.797	0.025	0.749	0.845	0.729	0.716	0.000**

AUC, area under curve; SE, standard error.

*P < 0.05.

**P < 0.01.

To compare the potential difference between MPP and some subtype NMPP groups, we divided the NMPP group into bacterial pneumonia (BP) and viral pneumonia (VP) and examined the relative differences in several criteria including aging, sex, hospital stays, fever, etc., which showed similar distinction from MPP group (Supplementary Table S1).

## 4 Discussion

**Figure 3:**
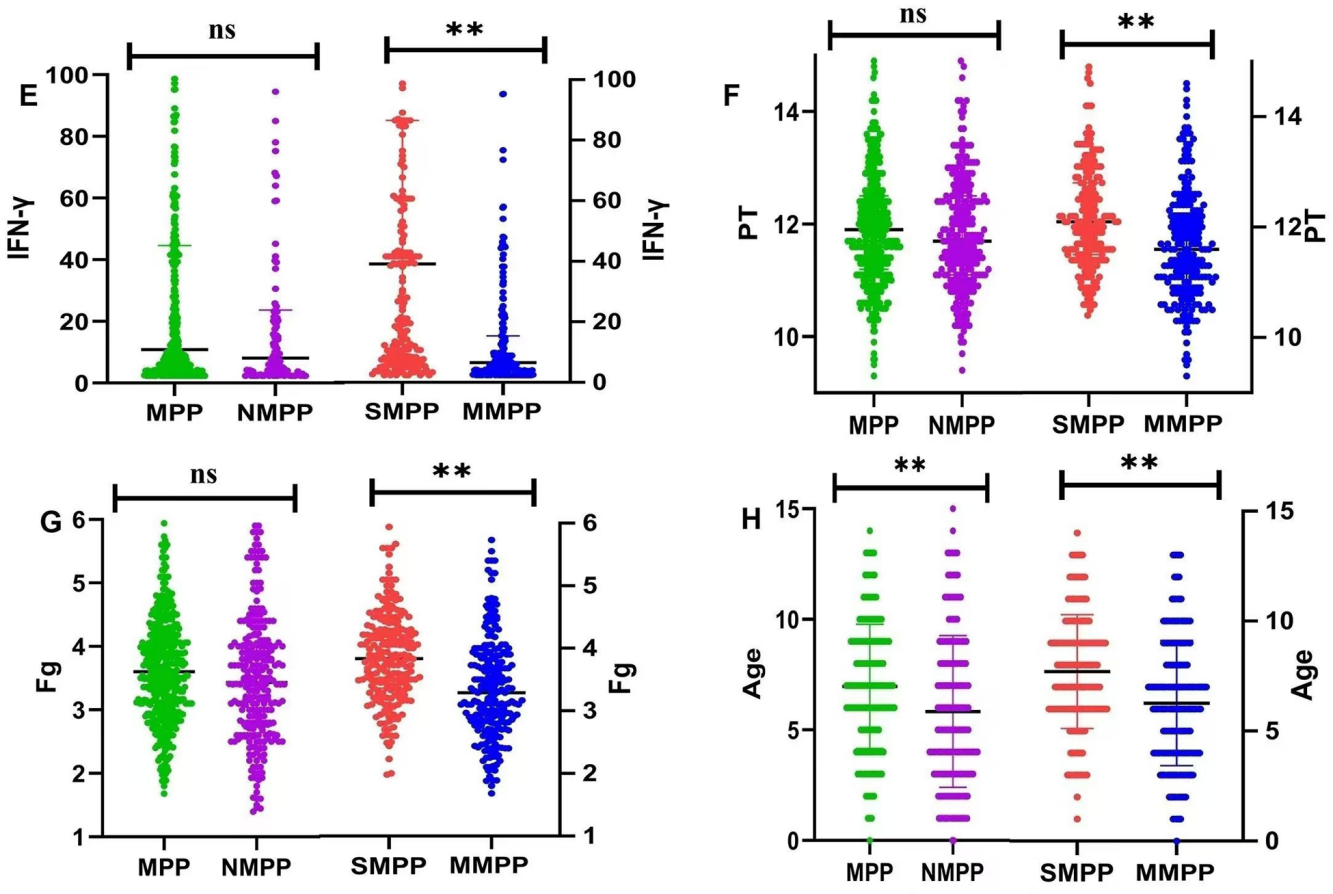
**(E)** differences of IFN-γ between MPP group and NMPP group, SMPP group and MMPP group; **(F)** differences of PT between MPP group and NMPP group, SMPP group and MMPP group; **(G)** differences of Fg between MPP group and NMPP group, SMPP group and MMPP group; **(H)** differences of Age between MPP group and NMPP group, SMPP group and MMPP group. Ns means no statistical difference, *p < 0.05, **p < 0.01.

This study evaluated a multitude of disease indicators or factors including general clinical characteristics, blood biochemistry, damage to extrapulmonary organs and immune response of patients. It comprehensively dissected the disparities in diverse indicators among different groups. Compared to NMPP group, MPP group was more frequently diagnosed in school-age children, and cough is more prominent in clinical manifestation (Table 1). Additionally, the SMPP group had significantly longer duration of fever and hospitalization as well as higher incidence of extrapulmonary complications than MMPP (Table 6, Figure 3), which was consistent with a report by Zhao et al. (14). The age of SMPP group was older than that of MMPP group, which was consistent with the previous reports (15, 16). This might be due to the maturation of immune function. The occurrence of SMPP group is associated with aberrant autoimmune response, which is more often observed in school-age children compared to preschool children (17). An excessive immune response to *M. pneumoniae* results in an overabundance of inflammatory cytokines, and ultimately development of SMPP. Some previous studies showed that MPP group had various extrapulmonary manifestations including meningitis, cerebral infarction, myocarditis, hepatitis, and so on (18, 19). Consistently, the extrapulmonary manifestations presented with liver function damage, myocardial damage, rash and abnormal coagulation index in our research. More serious extrapulmonary complications were not found, which might be attributed to timely and effective anti-*M. pneumoniae* treatment.

*M. Pneumoniae* infection can involve in the entire airway, even the interstitial lung and alveoli. Thus, the imaging findings of MPP may vary depending on the region of *M. pneumoniae* infection. From the perspective of chest imaging, there was no difference in the sites of the two types of pneumonia (MPP and NMPP groups). However, in our study, the incidence of pulmonary consolidation in children with MPP (12.3%) was higher than that in NMPP group (4.8%) (Table 1) and children in SMPP group (24.34%) was higher than that in MMPP group (0.84%) (Table 6). In a previous study with 393 hospitalized children diagnosed with MMP, the most common radiological finding was lobar or segmental consolidation (37%) (20). Clinically, *M. pneumoniae* infection is more likely to cause the development of lung tissue damage, such as pulmonary necrosis or pulmonary thrombosis. Hence, the SMPP group should be identified as early as possible in clinical work to prevent progression of disease. Therapeutically, to exclude the impacts of age and gender, analysis of covariance and Logical regression were used to account for the baseline differences in the probability between SMPP and MMPP group. It showed that the number of oxygen therapy cases were no obviously difference between the SMPP and MMPP group.

**Figure 4:**
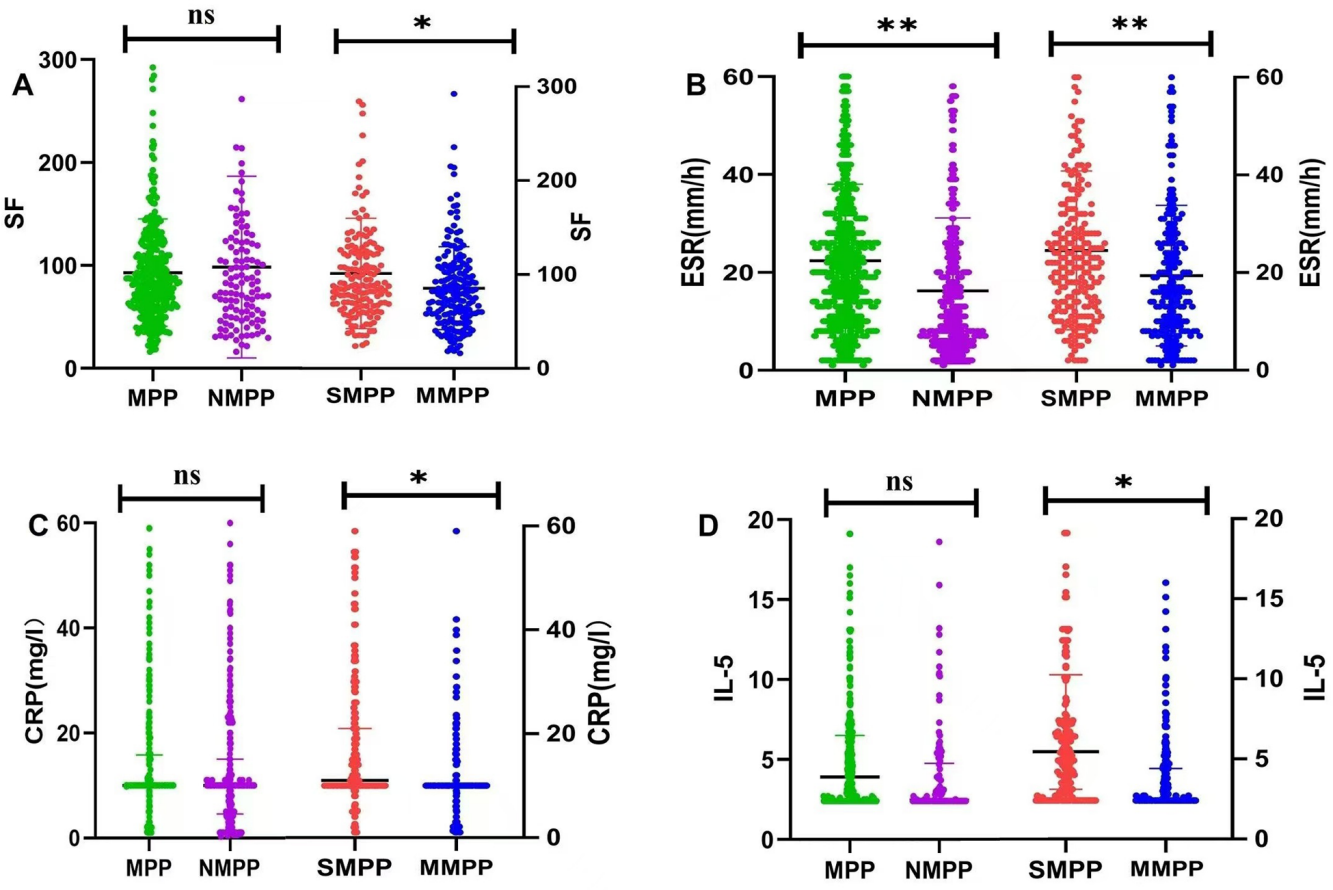
**(A)** differences of SF between MPP group and NMPP group, SMPP group and MMPP group; **(B)** differences of ESR between MPP group and NMPP group, SMPP group and MMPP group; **(C)** differences of CRP between MPP group and NMPP group, SMPP group and MMPP group; **(D)** differences of IL-5 between MPP group and NMPP group, SMPP group and MMPP group. Ns means no statistical difference, *p < 0.05, **p < 0.01.

*M. pneumoniae* infection can induce immune responses in the organism, resulting in immune damage and immune imbalance. PCT, PLT, HBP and ESR are hypersensitive markers during infection and inflammation. Fan et al. found that non-specific inflammatory indicators, such as the PCT, ESR levels in MPP group, were clearly higher (13). We found in our study, compared to the NMPP group, the levels of PLT, HBP and ESR levels in MPP group were much higher (Table 2). This suggests that the inflammatory response and tissue damage are aggravated during *M. pneumoniae* infection compared with NMPP group. Further, we found that the SF, ESR, and CRP measures were significantly higher in the SMPP group relative to the MMPP group (Table 7, Figure 4). Previous work reported that SF level may be useful as an indicator of the severity of pediatric MPP for initiation of corticosteroid therapy (21) and was apparent warning indicators for SMPP group described by Yuanyuan et al. (22, 23). Therefore, these inflammatory factors may be contribute to predict occurrence of SMPP. Nonetheless, no differences were observed in HBP, PCT and TNF between the two groups. Besides, WBC count is one of the most commonly inflammatory indicators in the clinical practice, but in our study there was no significant change in WBC count between the two groups, which is consistent with the study by Choi et al. (20). It suggests that WBC count cannot be used as an early warning indicator of SMPP.

Some researchers found that the cytokine-mediated inflammation and immune response in the host played an important role in mycoplasmal pathogenicity (24). Increasing evidence shows that monoclonal antibodies could substantially reduce or inhibit the adhesion of bacteria to human respiratory epithelial cells *in vitro* (25–27). In humans, IgG and IgM antibodies are present in the post-infection sera. Our study demonstrates that compared to NMPP group there was significant increase in IgG in the MPP group (Table 3). The results indicate that MPP group exhibit excessive immune response and immune dysfunction. The context of protective immunity against MPP group remains largely unknown in humans. Therefore, further large-scale studies are needed. Some studies suggest that cellular immune function changes are most pronounced, was associated with the development of interstitial pneumonia by *M. pneumoniae* infections (28). Our study also showed that both MPP group and SMPP group had significantly decreased serum CD3^+^ T cells compared with corresponding NMPP group and MMPP (Tables 3, 8). The above results revealed that CD3^+^ T cells and CD3^+^CD8^+^ T cell counts were decreased in SMPP group were likely associated with the development of SMPP group. Besides, *M. pneumoniae* infection activates the coagulation system through a variety of ways, leading to coagulation abnormalities and promotion of thrombosis. In the indices of blood coagulation, we found that MPP group had a lower level of TT than NMPP group. This result indicated that MPP group had a higher risk of embolism (Table 4).

In addition to direct infection by *M. pneumoniae* (29, 30), an excessive immune reaction in the host plays a crucial role in the development of MPP group (31). The imbalance of Th1/Th2 function after *M. pneumoniae* infection is an important immunological mechanism of MPP group (32, 33). Here, we analyzed the levels of EOS, IgE, IL-4, IL-5 and INF-γ in children with pneumonia. No difference was observed in the levels of serum IL-5 and INF-γ between MPP and NMPP groups (Table 5 and Figures 3, 4). However, the expression levels of serum IL-5 and INF-γ in the SMPP group were significantly higher than those in the MMPP group (Table 10 and Figures 3, 4).

After *M. pneumoniae* infects the human lower respiratory tract, its membrane lipoproteins are recognized and bound by the TLR on alveolar macrophages through MyD88-dependent pathway, which further enables the activation of NF-κB transcription factor to produce corresponding cytokines and chemokines, ultimately damaging airway mucosal epithelial cells and causing various complications (34, 35). The results of this study suggest that IFN-γ is one of the main cytokines involved in inflammatory response after mycoplasma infection. IFN-γ secreted by natural killer cells can improve the lysosomal activity of macrophages and stimulate macrophages to secrete pro-inflammatory factors to aggravate inflammation in the body (36, 37). Previous studies have suggested abnormal expression of Th1/Th2 cytokines in the peripheral blood of children with severe MPP. The occurrence of *M. pneumoniae* infection can promote the differentiation of Th2 cells, leading to IL-4, IL-5, IL-6, IL-10, and IL-13, and are responsible for inducing T cell dependent humoral immunity against extracellular pathogens. High expression of IL-5 in peripheral blood promote phagocyte activation, cellular killing and immune modulation (38–40). The excessive inflammation reaction may caused the release of cytokines, which might be related to the severity of SMPP group (Table 10). Therefore, we speculate that IL-5 and IFN-γ may contribute to disease severity and prognosis (Table 11 and Figures 2–4).

The logistic regression analysis confirmed that IL-5, INF-γ, SF, PT, Fg and ESR were independent risk factors for SMPP group. According to the results of ROC curves, when cutoff value is IL-5 ≥2.85 pg/ml and IFN-γ ≥10.05 pg/ml were good predictors for SMPP group, the sensitivity and specificity of IL-5 is 72.3%, 61.3% and the sensitivity and specificity of IFN-γ is 66.5%, 67.7%, 95% CI respectively are 0.648–0.764 and 0.653–0.767. The above results showed that the plasma IFN-γ and IL-5 both increased in the SMPP group, which showed good predictive efficacy for the occurrence of SMPP group and had great significance for the personalized diagnosis and treatment of MPP group (Table 11, Figures 2–4).

However, our study has the following limitations. This was a single-center study, and most of the hospitalized children had a history of macrolide antibiotics use before admission. We did not examine the resistance of macrolide drugs. Some patients included in the study might have co-infection with other pathogens that were not detected during hospitalization. The severity of patients’ disease was not evenly distributed, which had a certain impact on the experimental results. Meanwhile, the blood samples collected for analysis were not controlled at a uniform point in time. These impact factors may lead to some research bias and should be further confirmed in larger, multi-center, prospective studies in the future. To consider the potential difference between MPP group and some subtype NMPP group, we divided the NMPP group into bacterial pneumonia (BP) and viral pneumonia (VP) and examined the relative differences in several criteria including aging, sex, hospital stays, fever, etc., which showed similar distinction from MPP group (Supplementary Table S1). Further analysis of detailed mechanisms involved may be helpful for clinical practice.

In conclusion, we found that children with MPP group were more likely to have fever, extrapulmonary complications, abnormal coagulation function and higher inflammatory markers (PLT, HBP, ESR, PCT, CTnI and AST). These indicators included in our study were clinically important and appropriately indicated the immune response and were conducive to clinical practice. Furthermore, in order to find indicators that are helpful in evaluating diseases, especially warning indicators for SMPP, we conducted a comprehensively retrospective analyse of MPP from multiple dimensions such as clinical characteristics, imaging examinations, and corresponding laboratory parameters of patients. Our study confirmed that the determination of PT, Fg, SF, IL-5 and IFN-γ can be practical markers for SMPP group. This study may help pediatricians to accomplish early identification of *M. pneumoniae* infection and assess its severity. Further studies conducted in multiple centers are needed to validate our research.

## Data Availability

The raw data supporting the conclusions of this article will be made available by the authors, without undue reservation.
