# Supplementary material for: Clinical characteristics and risk factors for Mycoplasma pneumoniae pneumonia in children
Source: Front Pediatr. 2024 Dec 23;12:1438631. doi: 10.3389/fped.2024.1438631 (PMC11701002; doi:10.3389/fped.2024.1438631)
Supplement: Supplementary file 1 [file Table1.docx]

**Supplement Table 1:** Comparison of demographic and clinical characteristics between MPP and BPP, VPP groups after analysis of covariance and Logical regression

| **General information** | **MPP (464)** | **BP (219)** | **VP (74)** | **P** |
| --- | --- | --- | --- | --- |
| **Sex(male/female)** | 215/249 | 120/99 | 40/34 | 0.085 |
| **Age(years)** | 6.97 ± 2.81 | 6.00 ± 3.60 | 5.42 ± 2.81 | 0.000** |
| **Fever duration (days)** | 6.1 ± 3.37 | 4.85 ± 3.26 | 5.23 ± 3.53 | 0.000** |
| **Hospital stays (days)** | 6.23 ± 2.16 | 5.97 ± 2.25 | 5.81 ± 1.58 | 0.378 |
| **Fever, n(%)** | 425 (91.59) | 191 (87.21) | 60 (81.10) | 0.003 |
| **Cough, n(%)** | 444 (95.69) | 187 (85.39) | 63 (85.12) | 0.000** |
| **Wheezing, n(%)** | 13 (2.80) | 8 (3.65) | 2 (2.70) | 0.771 |
| **Extrapulmonary symptom, n(%)** | 28 (6.03) | 6 (2.74) | 1 (1.35) | 0.032* |
| **Pulmonary consolidation, n (%)** | 57 (12.3) | 14 (6.40) | 0 (0.00) | 0.002** |

Notes: The data were the baseline measurement results made within 24 h after admission. P < 0.05 indicates statistical significance.

Abbreviations: BP, Bacterial pneumonia; VP, Viral pneumonia.
